# International trade, dietary change, and cardiovascular disease health outcomes: Import tariff reform using an integrated macroeconomic, environmental and health modelling framework for Thailand

**DOI:** 10.1016/j.ssmph.2019.100435

**Published:** 2019-07-31

**Authors:** Henning Tarp Jensen, Marcus R. Keogh-Brown, Bhavani Shankar, Wichai Aekplakorn, Sanjay Basu, Soledad Cuevas, Alan D. Dangour, Shabbir H. Gheewala, Rosemary Green, Edward Joy, Nipa Rojroongwasinkul, Nalitra Thaiprasert, Richard D. Smith

**Affiliations:** a London School of Hygiene & Tropical Medicine, UK; b University of Copenhagen, Denmark; c SOAS University of London, UK; d Ramathibodi Hospital, Mahidol University, Thailand; e Stanford University, USA; f King Mongkut's University of Technology Thonburi (KMUTT), Thailand; g Institute of Nutrition, Mahidol University, Thailand; h Chiang Mai University, Thailand; i University of Exeter, UK

**Keywords:** International trade, Diet, Import tariffs, CGE, Simulation

## Abstract

United Nations (UN) member states have, since 2011, worked to address the emerging global NCD crisis, but progress has, so far, been insufficient. Food trade policy is recognised to have the potential to impact certain major diet-related health and environmental outcomes. We study the potential for using import tariff protection as a health and environmental policy instrument. Specifically, we apply a rigorous and consistent Macroeconomic-Environmental-Demographic-health (MED-health) simulation model framework to study fiscal food policy import tariffs and dietary change in Thailand over the future 20 year period 2016-2035. We find that the existing Thai tariff structure, by lowering imports, lowers agricultural Land Use Change (LUC)-related GHG emissions and protects against cholesterol-related cardiovascular disease (CVD). This confirms previous evidence that food trade, measured by import shares of food expenditures and caloric intakes, is correlated with unhealthy eating and adverse health outcomes among importing country populations. A continued drive towards tariff liberalization and economic efficiency in Thailand may therefore come at the expense of reduced health and environmental sustainability of food consumption and production systems. Due to large efficiency losses, the existing tariff structure is, however, not cost-effective as an environmental or health policy instrument. However, additional simulations confirm that stylized 30% food sector import tariffs generally improve nutritional, clinical health, demographic, and environmental indicators across the board. We also find that diet-related health improvements can go hand-in-hand with increased Saturated Fatty Acid (SFA) intakes. Despite limited cost-effectiveness, policy makers from Thailand and abroad, including WHO, would therefore be well advised to consider targeted fiscal food policy tariffs as a potential intervention to maintain combined health and environmental sustainability, and to reconsider the specification of WHO dietary guidelines with their focus on SFA intake (rather than composition of fatty acid intake) targets.

## Highlights

• Fully integrated Macroeconomic-Environmental-Demographic-health model for Thailand.
• Methodologically rigorous quantitative approach to analysing trade and health.
• Existing Thai tariff structure protects against cholesterol-related CVD illness.
• Food import tariffs generally improve nutritional, health and demographic outcomes.
• Protective import tariffs generally lead to economic-environmental trade-offs.

## 1 Introduction

The political need to address growing diet-related health problems at the global level has recently received widespread recognition. In September 2011, United Nations (UN) member states, gathering at the first UN High-Level Meeting on non-communicable diseases (NCDs), accepted, for the first time, that a global NCD crisis was emerging (UN, 2011). At that point, the World Health Organization (WHO) estimated that 36 million deaths, out of a total 57 million global deaths, were due to NCDs, and that nearly 80 percent of NCD deaths were occurring in developing countries (ibid.). The crisis has also been characterized as “a barrier to development goals including poverty reduction, health equity, economic stability, and human security” (Beaglehole, 2011). The 66th World Health Assembly subsequently endorsed the WHO Global Action Plan for the Prevention and Control of Non-communicable Disease 2013–2020 (WHO, 2013).

In 2015, the attention of the NCD community turned to the newly adopted UN Sustainable Development Goals (SDGs) and SDG 3.4: “By 2030, reduce by one third premature mortality from NCDs through prevention and treatment and promote mental health and well-being” (UN, 2015). However, in anticipation of the third UN High-Level Meeting on NCDs in 2018, the WHO NCD Progress Monitor 2017 report concluded that “Progress … has been insufficient and highly uneven” and “… the current rate of decline in premature death from NCDs will not meet the SDG target” (WHO, 2017a). The WHO Global Action Plan established six objectives and identified a list of 16 cost-effective interventions, the so-called ‘best buys’ (WHO, 2013). An updated list of interventions was published in 2017 (WHO, 2017b). While the new list of (cost-effective) interventions to reduce modifiable risk factors for NCDs (Objective 3) included excise taxes to reduce tobacco use and harmful use of alcohol, no tax interventions were proposed for improving unhealthy diets.

In this paper, we investigate how trade protection, through imposition of import tariffs, may affect incidence and prevalence of NCD in the case of Thailand – a middle-income country which is currently undergoing a nutritional transition and where the burden of NCDs are growing dramatically. Specifically, we apply a newly constructed MED-health model for Thailand (Jensen et al., 2019) to analyse the impact of the existing protective import tariff structure and to study the general policy impact of imposing new protective food import tariffs in the fight to control rising cholesterol-related cardiovascular disease (CVD) in a middle-income and nutritional transition setting.

Recent Thai government data suggests that NCDs have been responsible for more than 75% of all Thai deaths over the past decade, and that premature death rates have been trending upwards during 2012–2015 for the four major NCDs: cerebrovascular disease (33.4–40.9 per 100,000 population), ischemic heart disease (22.4–27.8 per 100,000 population), diabetes (13.2–17.8 per 100,000 population), and chronic obstructive pulmonary disease (3.8–4.5 per 100,000 population) (MoPH, 2017). In parallel, key CVD risk factors have increased dramatically over the past decade (2005–2015): rates of overweight (BMI>25.0 kg/m^2^; from 16.1% to 30.5%) and rates of obesity (BMI>30.0 kg/m^2^; from 3.0% to 7.5%). Additional WHO estimates indicate that ischaemic heart disease and stroke increased during 2000–2012, and that they constituted the two largest contributors to Thai mortality in 2012 accounting for respectively 68,800 (13.7%) and 51,800 (10.3%) deaths (WHO, 2015). While the Thai NCD share of deaths is around the global average of 70% (WHO, 2017a), the growing trends are alarming. It is therefore critical to address the emerging NCD and CVD crisis in Thailand.

The MED-health model for Thailand which we employ (Jensen et al., 2019) is constructed on the basis of a trade-focused macroeconomic Computable General Equilibrium (CGE) model framework (Devarajan, Lewis, & Robinson, 1990; Robinson, 1991, pp. 885–947). This so-called ‘Standard Model’ framework is fully documented in Löfgren, Lee Harris, and Robinson (2002) and comes with a fully specified set of government indirect tax instruments including import tariffs. It is therefore ideal for analysing the impact of trade liberalization and trade protection on health outcomes. Moreover, the multi-sector nature of the model allows us to analyse the impact of protective tariffs on individual food sectors and across all commodity sectors.

The model also captures a key NCD *health pathway*, whereby changes in consumption of fatty acids from food commodities cause cholesterol-related CVD illness. This makes the model particularly useful for Thai health policy analysis. While Thai policy makers have established NCD targets according to WHO guidelines (MoPH, 2017), they have not yet implemented recommended Saturated Fatty Acid measures to address unhealthy diets (WHO, 2017a). Our analysis, with its focus on fatty acid composition and cholesterol build-up, therefore aims to fill a void in the Thai policy envelope. Finally, our model also includes a simplified Land Use Change (LUC) module to measure environmental outcomes. The multi-dimensional nature of our model framework thereby allows us to focus on trade-offs between health, economic, and environmental outcomes, and to provide a broad holistic assessment of the cost-effectiveness of employing import tariffs as a public health intervention tool.

Import tariffs have, historically, been employed to protect infant industries and generate critical tax revenues in low-income countries. Since the debt crisis in the mid-1980s and the subsequent development of Structural Adjustment Programs (SAP), trade liberalization and tariff reduction have, however, been the norm for stabilizing middle-income economies and promoting economic growth (the ‘Washington Consensus’). The drive towards reducing tariff barriers has also been enhanced by the establishment of the World Trade Organization (WTO), in 1995, and the accompanying WTO regulatory framework which, generally, prohibits discrimination between trading partners. While the trade liberalization agenda has recently come under pressure from the “America First” strategy, favoured by the current US presidency, the majority of the global community continues to support the global free trade agenda.

At the same time, the health argument has received relatively little attention in the trade liberalization debate. The critical importance of taking a macroeconomic perspective on the prevention of NCDs has been forcefully argued (Smith, 2012). Nonetheless, the economic CGE literature on (agricultural) tariff liberalization, which is broad and includes both single-country studies (De Melo, 1988) and, since the late 1990s, multi-country studies of regional trade agreements (Gilbert, 2008; Robinson & Thierfelder, 2002), only contains one published (single country) study with a health focus (Cockburn, Emini, & Tiberti, 2014), and the latter Cameroon study, which focuses on the nutritional child ‘caloric poverty’ impact of potential food tariff exemptions in the aftermath of the global economic crisis, does not model clinical health outcomes.

The nascent quantitative literature on trade and health, which has emerged over the past 10 years, covers additional descriptive and statistical designs ranging from simple cross-country correlation analyses of unhealthy and imported food expenditure shares (Estimé, Lutz, & Strobel, 2014) and difference-in-difference evaluation studies of natural experiments of bilateral Free Trade Agreements and WTO accessions (Baker, Friel, Shram, & Labonte, 2016; Barlow, Mckee, Basu, & Stuckler, 2017; Schram et al., 2015) to structural statistical approaches to investigate possible mechanisms of broader relationships (Baker et al., 2016). The literature is, however, marred by problems of poorly defined exposures and mechanisms not sufficiently explored, and there continues to be a need for “more methodologically rigorous and consistent approaches in future quantitative studies” (Cowling, Thow, & Porter, 2018). Our study aims to fill this void by applying a fully integrated quantitative MED-health simulation model with an explicit and clearly defined cholesterol-related CVD-focused health pathway where nutritional exposure is governed by household-specific Almost Ideal Demand Systems (AIDS), where our Total:HDL ratio cholesterol biomarker is governed by a validated structural relationship with fatty acid intake shares (Mensink, Zock, Kester, & Katan, 2003), and where clinical health outcomes, as well as pecuniary health cost and labour market feedback effects, are derived from rigorous modelling (Jensen et al., 2019). Structural details of our MED-health model framework are provided next.

## 2 Materials and methods

### 2.1 Simulation model

**Fig. 1:**
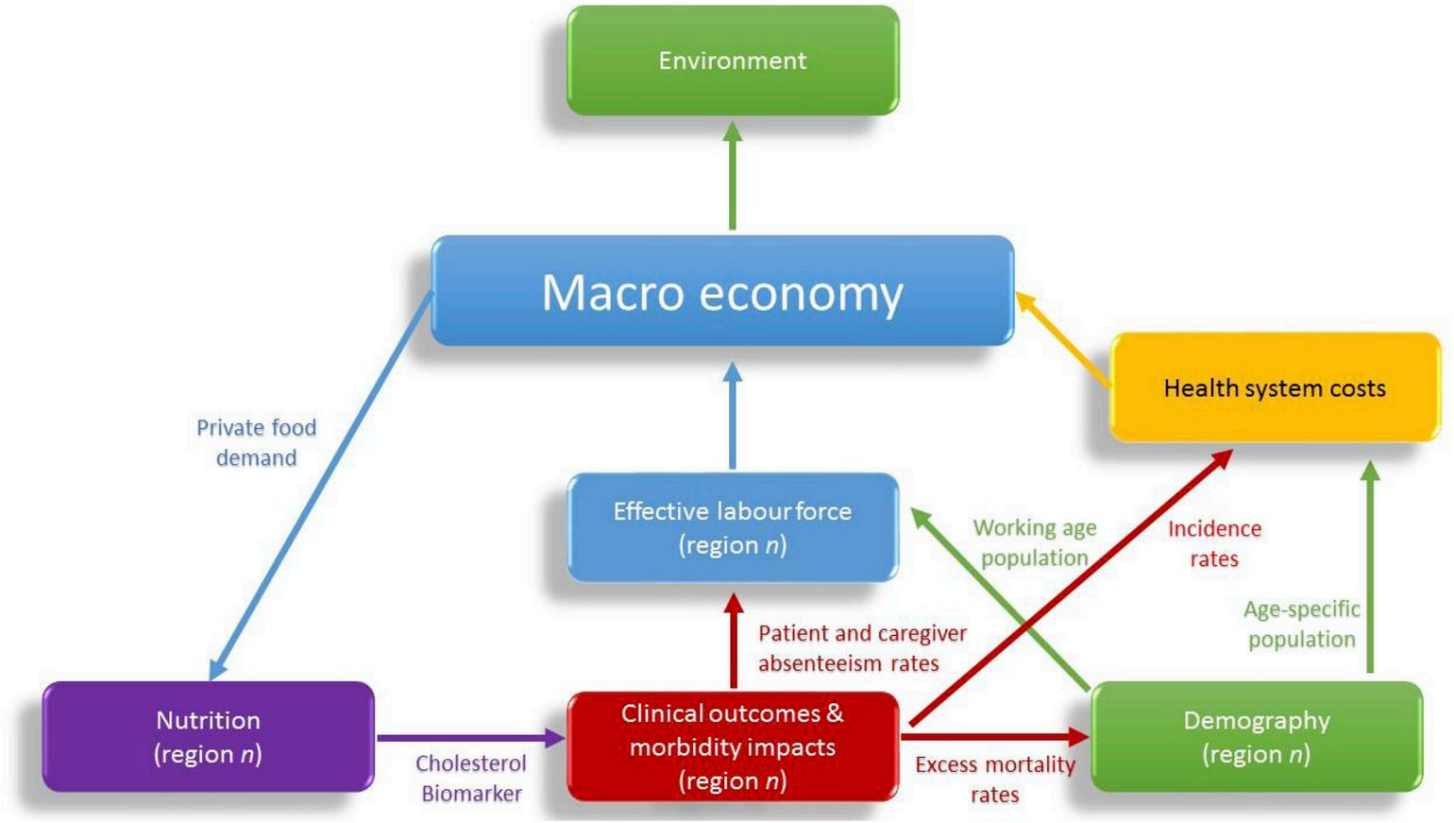
**MED-health model framework and feedback effects between the macroeconomy and regional sub-models**.

The Thai MED-health model framework for cholesterol-related CVD illness which we employ is a fully integrated recursively-dynamic model for 2016–2035 covering fully integrated models and modules for economic, nutritional, clinical health, and demographic outcomes, and a satellite module for environmental outcomes. The pathways of the fully integrated model framework are illustrated in Fig. 1. The key feature is that economic incentives from the macroeconomic model determine regional food demand and nutritional intakes in the nutrition module and, via serum cholesterol biomarker build-up, impact health outcomes in the clinical health outcome module (producing distributions of illness-specific incidence and mortality rates). The clinical health impacts, subsequently, affect regional effective labour force participation rates (through working-age patient and caregiver time losses) and regional population distributions (through patient mortality) in the Demographic module. Morbidity and demographic outcomes, finally, interact to produce labour force and health cost impacts which feed back into the macroeconomic CGE model. The model has been previously documented in Jensen et al. (2019). Detailed model structures are presented, below, for completeness.

### 2.2 Macroeconomic CGE model

The macroeconomic CGE model is a dynamically-recursive extension to the ‘Standard model’ which is fully documented in Löfgren et al. (2002). Dynamic model extensions include labour and capital factor updating equations, while regional land factor supplies were assumed to be fixed. The core CGE model is calibrated to a 2007 Social Accounting Matrix (SAM), the most recent Thai SAM available at the time of model construction (NESDB, 2015). The SAM contains seven production factors including four regional land types, unskilled and skilled labour, and capital, where skilled/unskilled labour and land/capital value added breakdowns were based on Global Trade Analysis Project (GTAP) data (GTAP, 2017). In order to allow for regional modelling, the SAM was further extended to include nine representative regional household types (Bangkok and rural-urban splits of south, central, north, and northeast regions) derived from the 2011 Household Socio-Economic Survey (NSO, 2008).

Household demand is governed by household-specific Almost Ideal Demand Systems (AIDS) (for details, see below); Production is specified as Constant Elasticity of Substitution (CES) functions of aggregate intermediate input demands (individual commodity input demands are determined by Leontief specifications) and aggregate factor input demands (individual factor input demands are also determined by CES specifications) with standard elasticity values for the top-level production specifications (0.8) and the bottom-level factor input demand specifications (0.6); Trade between domestic and foreign agents is specified as a function of relative prices (determined by the real exchange rate), based on Armington CES specifications on the import side and Constant Elasticity of Transformation (CET) specifications on the export side. Standard trade elasticity values were applied on the import side (0.8) and on the export side (1.6). Our modelling of production, consumption, and trade covers 49 sectors (including six primary food, and five processed food and beverage commodity types),^1^ but we restrict ourselves to present results for eight aggregate sectors (including one primary food, and four processed food and beverage sectors – see Table 1) in order to keep our analyses focussed.

**Table 1:** **Structure of Economic CGE model**.

	Import Share	Export Share	Import Tariffs	Sales Share	Household Share
primary food sectors	5.3%	3.4%	1.4%	3.5%	4.9%
other primary sectors	4.5%	6.1%	0.3%	2.2%	1.2%
palm cooking oil	3.1%	11.8%	0.0%	0.2%	0.4%
other edible oils	28.9%	20.9%	0.2%	0.1%	0.1%
other processed foods	10.4%	43.1%	2.8%	5.9%	8.0%
beverages	14.5%	9.8%	22.7%	1.8%	5.8%
Other manufacturing	39.1%	67.9%	1.6%	47.1%	28.1%
Services	11.2%	13.8%	0.0%	39.3%	51.6%
Total/Average	24.0%	35.5%	1.6%	100.0%	100.0%

*Source*: 2007 Thai Social Accounting Matrix (NESDB, 2015).

Subsequently, we used historical Thai GDP growth rates (WB, 2015a, WB, 2015b) to establish 2015 as base year for our 2016–35 policy simulations. Our counterfactual 2016-35 growth path was, similarly, based on historical real (3.9% p.a.) and nominal (6.2% p.a.) Thai GDP growth rates for 1998–2014 (ibid.), and on a balanced macro closure with a fixed government consumption-to-absorption ratio.

The structure of trade, domestic sales, and household consumption in the economic model is set out in Table 1: The share of imports in domestic sales (Import Share), the share of exports in domestic production (Export Share), import tariff rates (Import Tariffs), the share of each sector in domestic sales (Sales Share), and the share of each sector in household consumption (Household Share). In this study, we report results for eight aggregate sectors including five food groups: one primary food and four processed food commodity types. Two edible oil sectors are distinguished due to the importance of oil palm production in Thailand (Jensen et al., 2019). The numbers indicate that Thailand is a fairly open economy with average import and export shares of respectively 24.0% and 35.5%. Trade shares are particularly high for “other manufacturing” (manufactured goods other than processed foods), and “other processed foods” (processed foods other than edible oils) and “other edible oils” (mainly soybean oil). Trade shares are lower for the highly taxed “beverages” sector, and very low for the palm cooking oil sector which is protected by non-tariff barriers (NTBs). Baseline import tariff rates are fairly low (averaging 1.6%) except for the beverages sector which is protected by a fairly high 22.7% tariff rate. Finally, the sales structure reflects that Thailand is a middle-income country in transition to becoming a service-dominated society. Domestic sales are dominated by manufactured goods (55.0%), while primary and tertiary service sector sales account for respectively 5.7% and 39.3%.

The remaining three fully integrated modules were stratified in the same way as our CGE model, i.e. including nine representative household types, thereby allowing our import tariff protection strategies to cause differential region-specific dietary exposures and to have differential nutritional, health, demographic, and welfare impacts. In the following five subsections, we describe the three remaining fully integrated modules, one by one, the feedback effects from our fully integrated modules to the macroeconomic CGE model, and our simplified environmental satellite module.

### 2.3 Dietary exposure and nutritional transmission module

For each of our nine households (h∈H), dietary exposure is governed by household-specific AIDS demand systems covering 49 commodities (c∈C) which maps to the eight aggregate commodities presented in Table 1, above, and reported in the Results section, below. Household-specific consumption of commodity c by household h at time t ($x_{c,h,t}$) is determined by household-specific consumption shares ($w_{c,h,t}$) and disposable income ($eh_{h,t}$), and consumption shares are governed by first order conditions for cost minimization:

(1a) $$x_{c,h,t}=\frac{w_{c,h,t}eh_{h,t}}{p_{c,t}},\;\;\;\;c\in C,\;h\in H,\;t\in T$$

(1b) $$w_{c,h,t}=\bar{\alpha_{c,h}^{AIDS}}+\bar{\beta_{c,h}^{AIDS}}\log\left(\frac{eh_{h,t}}{P_{t}}\right)+\underset{cp\in C}{\sum }\bar{\gamma_{c,cp,h}^{AIDS}}\text{log}\left(p_{cp,t}\right),\;\;\;\;\;c\in C,\;h\in H,\;t\in T$$

where $\bar{\alpha_{c,h}^{AIDS}},\;\bar{\beta_{c,h}^{AIDS}},\bar{\gamma_{c,cp,h}^{AIDS}}$: AIDS demand system parameters, $p_{c,t}$: commodity-specific consumer prices, $P_{t}$: GDP deflator price index. Parametrization of the AIDS demand systems was informed by Thai-specific income and uncompensated price elasticities (Lippe & Isvilanonda, 2010; Suebpongsakorn, 2008) and non-Thai edible oil cross-price elasticities from the literature (Kim & Chern, 1999; Yen & Chern, 1992), and based on standard price and income elasticity formulas (Green & Alston, 1990, 1991).

It is well-known that the composition of fatty acid intakes governs the build-up of the Total:HDL cholesterol biomarker (Mensink et al., 2003). Nutritional outcomes are therefore measured in terms of energy intake shares from Saturated Fatty Acids ($e_{h,t}^{SFA}$), Mono-Unsaturated Fatty Acids ($e_{h,t}^{MUFA}$), and Poly-Unsaturated Fatty Acids ($e_{h,t}^{PUFA}$). Household-specific consumption patterns ($x_{c,h,t}$) determine fatty acid energy intake shares in equations (2a), (2b), (2c), and these, in turn, determine household-specific average cholesterol biomarker build-up ($\Delta chol_{h,t}$) in equation (3):

(2a) $$e_{h,t}^{SFA}=\frac{\sum_{c\in C}\bar{\alpha_{c,h}^{SFA}}x_{c,h,t}}{\sum_{c\in C}\bar{\alpha_{c,h}^{Total}}x_{c,h,t}},\;\;\;\;h\in H,\;t\in T$$

(2b) $$e_{h,t}^{MUFA}=\frac{\sum_{c\in C}\bar{\alpha_{c,h}^{MUFA}}x_{c,h,t}}{\sum_{c\in C}\bar{\alpha_{c,h}^{Total}}x_{c,h,t}},\;\;\;\;h\in H,\;t\in T$$

(2c) $$e_{h,t}^{PUFA}=\frac{\sum_{c\in C}\bar{\alpha_{c,h}^{PUFA}}x_{c,h,t}}{\sum_{c\in C}\bar{\alpha_{c,h}^{Total}}x_{c,h,t}},\;\;\;\;h\in H,\;t\in T$$

(3) $$\Delta chol_{h,t}=\bar{\alpha_{c,h}^{chol,SFA}}\Delta e_{h,t}^{SFA}+\bar{\alpha_{c,h}^{chol,MUFA}}\left(\frac{e_{h,t}^{MUFA}}{e_{h,t}^{Total}}\right)\Delta e_{h,t}^{MUFA}+\bar{\alpha_{c,h}^{chol,PUFA}}\Delta e_{h,t}^{PUFA},h\in H,\;t\in T$$

where ($\bar{\alpha_{c,h}^{SFA}},\bar{\alpha_{c,h}^{MUFA}},\bar{\alpha_{c,h}^{PUFA}}$): SFA, MUFA and PUFA fatty acid energy contents of commodity c; $\bar{\alpha_{c,h}^{Total}}$: total energy contents of commodity c; ($\bar{\alpha_{c,h}^{chol,SFA}},\bar{\alpha_{c,h}^{chol,SFA}},\bar{\alpha_{c,h}^{chol,SFA}}$): cholesterol biomarker build-up coefficients. Nutritional coefficients for individual food commodity groups ($\bar{\alpha_{c,h}^{SFA}},\bar{\alpha_{c,h}^{MUFA}},\bar{\alpha_{c,h}^{PUFA}},\bar{\alpha_{c,h}^{Total}}$) were based on information from the 2004–2005 National Thai Food Consumption Survey (Jitnarin et al., 2010; Kosulwat et al., 2006) and the 2011 Household Socio-Economic Survey (NSO, 2014), while the link between household-specific nutritional and cholesterol biomarker outcomes in equation (3) relies on statistical estimates of ($\bar{\alpha_{c,h}^{chol,SFA}},\bar{\alpha_{c,h}^{chol,SFA}},\bar{\alpha_{c,h}^{chol,SFA}}$) from Mensink et al. (2003).

Initial levels, frequencies, and distributions of household-specific cholesterol biomarkers were derived from the 2008–2009 Thailand National Health and Examination Survey (Aekplakorn et al., 2011; NHESO, 2009). Levels were used to initialize household-specific average cholesterol biomarker levels ($chol_{h,t_{0}}$). In addition, biomarker frequencies, covering 10 intervals (s∈STRATA) with equidistant end-points over the possible Total:HDL serum cholesterol ratio biomarker range [2.0; 7.0], were used to initialize household-specific biomarker distributions ($chol_{h,s,t_{0}}^{strata}$) and allowed us to measure changes in biomarker distributions by shifting distributions by the mean in equation (4):

(4) $$chol_{h,s,t}^{strata}=chol_{h,s,t-1}^{strata}+\Delta chol_{h,t},\;\;\;\;\;\;\;\;\;\;h\in H,\;s\in STRATA,\;t\in T$$

### 2.4 Clinical health module

In order to measure clinical health impacts, we simulated 11 equidistant sets of lookup tables, covering the 10 above-mentioned STRATA-intervals and further stratified across gender ((g∈G  = {male, female}), age (a∈A={0−4,5−9,…,65−69,70+}), and rural-urban locations (l∈L  = {rural, urban}), based on modelling of relative hazards for key events including non-fatal MI (MI-nf), non-fatal stroke (S-nf), fatal MI (MI-f), and fatal stroke (S-f), using an established empirical methodology (Lim et al., 2007) and relying on previously established log relative risks (Lewington et al., 2007). For each set of clinical illness outcomes (i∈I  = {MI-nf, S-nf, MI-f, S-f}), we subsequently used the lookup tables to derive detailed age-, gender-, and rural-urban location-specific 10th degree fitted polynomial coefficients ($\beta_{i,g,a,l,r}^{clin},\;r=0,\ldots ,10$) for predicting stratified clinical outcome rates ($clin_{h,s,\;g,a,i,t}^{rate}$) and, in turn, clinical outcome levels ($clin_{h,s,\;g,a,i,t}^{level}$) via multiplication with population strata ($POP_{h,\;g,a,t}$), in equations (5a), (5b):

(5a) $$clin_{h,s,g,a,i,t}^{rate}=\overset{10}{\underset{r=0}{\sum }}\underset{l|h}{\sum }\beta_{g,a,l,i,r}^{clin}{\left(chol_{h,s,t}^{strata}\right)}^{r},h\in H,\;s\in STRATA,g\in G,a\in A,i\in I,\;t\in T$$

(5b) $$clin_{h,\;g,a,i,t}^{level}=\underset{s\in STRATA}{\sum }\bar{freq_{h,s}^{strata}}*clin_{h,s,\;g,a,i,t}^{rate}*POP_{h,\;g,a,t},h\in H,g\in G,a\in A,i\in I,\;t\in T$$

where $\bar{freq_{h,s}^{strata}}$: household-specific population frequency distributions of cholesterol biomarker strata.

### 2.5 Demographic module

Our household-specific demographic modules are stratified across the same age groups ((a∈A), and gender ((g∈G) and regional household ((h∈H) strata, defined above, and used to predict births ($Births_{h,g,t}$), deaths ($Deaths_{h,g,a,t}$), net emigration ($Migr_{h,g,a,t}$), and population demographics ($POP_{h,\;g,a,t}$), based on household-specific transition probabilities ($\bar{p_{h,g,a,t}^{trans}}$), in equations (6a), (6b), (6c), (6d), (6e), (6f):

(6a) $$Births_{h,g,t}=\bar{sexratio_{g}}*\underset{\begin{array}{c} 15<a<49\\ g=female \end{array}}{\sum }\bar{asfr_{a,t}}*POP_{h,\;gp,a,t-1},h\in H,g\in G,\;t\in T$$

(6b) $$Deaths_{h,g,a,t}=\mu_{h,g,a,t}^{all}*POP_{h,\;a,g,t-1},h\in H,g\in G,a\in A,\;t\in T$$

(6c) $$Migr_{h,g,a,t}=\bar{\alpha_{h,g,a,t}^{MIGR}}*\left(1-\mu_{h,g,a,t}^{all}\right)*POP_{h,\;g,a,t-1},h\in H,g\in G,a\in A,\;t\in T$$

(6d) $$POP_{h,g,a,t|a=00-04}=\left(1-\bar{p_{h,g,a,t}^{trans}}\right)*\left(1-\bar{\alpha_{h,g,a,t}^{MIGR}}\right)*\left(1-\mu_{h,g,a,t}^{all}\right)*POP_{h,g,a,t-1}+Births_{h,g,t}\;\;\;\;h\in H,g\in G,\;t\in T$$

(6e) $$\begin{array}{l} POP_{h,\;g,a,t|a>00-04\;and\;a<70+}=\bar{p_{h,g,a-1,t}^{trans}}*\left(1-\bar{\alpha_{h,g,a-1,t}^{MIGR}}\right)*\left(1-\mu_{h,g,a-1,t}^{all}\right)*POP_{h,\;g,a-1,t-1}+\left(1-\bar{p_{h,g,a,t}^{trans}}\right)*\left(1-\bar{\alpha_{h,g,a,t}^{MIGR}}\right)*\left(1-\mu_{h,g,a,t}^{all}\right)*POP_{h,\;g,a,t-1},h\in H,g\in G,a\in A\backslash \left\{0-4,70+\right\},\;t\in T \end{array}$$

(6f) $$\begin{array}{l} POP_{h,\;g,a,t|a=70+}=\bar{p_{h,g,a-1,t}^{trans}}*\left(1-\bar{\alpha_{h,g,a-1,t}^{MIGR}}\right)*\left(1-\mu_{h,g,a-1,t}^{all}\right)*POP_{h,\;g,a-1,t-1}+\left(1-\bar{\alpha_{h,g,a,t}^{MIGR}}\right)*\left(1-\mu_{h,g,a,t}^{all}\right)*POP_{h,\;g,a,t-1},h\in H,g\in G,t\in T \end{array}$$

where $\bar{sexratio_{g}}$: sex ratio at birth; $\bar{asfr_{a,t}}$: age-specific fertility rates; $\mu_{h,g,a,t}^{all}$: all-cause mortality rates; $\bar{\alpha_{h,g,a,t}^{MIGR}}$: net emigration rates; $\bar{p_{h,g,a,t}^{trans}}$: population transition probabilities between age segments a and a+1. A set of 2010–35 Thai regional population projections (NESDB, 2013a; NESDB, 2013b), combined with age- and gender-specific sex ratios, fertility rates, and all-cause mortality rates, from the 2015 Revision of UN population projections (UN, 2015), were used to initialize the module, and demographic model calibration was completed through dynamic calibration of time-specific transition probabilities.

Our modelling of cause-specific cholesterol-related clinical outcome rates ($clin_{h,s,g,a,i,t}^{rate}$) in equation (5a) allows us to model (nutritional) feedback effects on all-cause mortality rates ($\mu_{h,g,a,t}^{all}$) from average illness-specific cholesterol-related excess mortality rates (, in equations (7a), (7b)$\mu_{h,g,a,i,t}^{excess})$:

(7a) $$\mu_{h,g,a,t}^{all,\;policy}=\mu_{h,g,a,t}^{all,count}+\underset{i\in \left\{MI-f,S-f\right\}}{\sum }\left(\mu_{h,g,a,i,t}^{excess,policy}-\mu_{h,g,a,i,t}^{excess,count}\right),h\in H,g\in G,a\in A,\;t\in T$$

(7b) $$\mu_{h,g,a,i,t}^{excess}=\underset{s\in STRATA}{\sum }\bar{freq_{h,s}^{strata}}*clin_{h,s,\;g,a,i,t}^{rate},h\in H,g\in G,a\in A,\;i\in \left\{MI-f,S-f\right\},\;t\in T$$

where $\mu_{h,g,a,t}^{all,count},\mu_{h,g,a,t}^{all,policy}$: all-cause mortality rates derived from counterfactual and policy simulations; $\mu_{h,g,a,i,t}^{excess,\;count},\mu_{h,g,a,i,t}^{excess,policy},i\in \left\{MI-f,S-f\right\}$: illness-specific cholesterol-related excess mortality rates predicted in counterfactual and policy simulations. Our all-cause mortality specification, equation (7a), assumes that the counterfactual all-cause mortality rates ($\mu_{h,g,a,t}^{all,\;count}$) encompasses the sum of the counterfactual excess mortality rates ($\underset{i\in \left\{MI-f,S-f\right\}}{\sum }\mu_{h,g,a,i,t}^{excess,count}$), without double-counting occurring due to multiple diagnoses arising.

### 2.6 Economic feedback effects and outcome measures

Based on the above modelling of nutritional transmission, working age population demographics ($POP_{h,\;g,a,t}$), and clinical health outcome levels ($clin_{h,s,g,a,i,t}^{level}$), we can measure additional non-pecuniary outcomes including caregiver leisure time losses ($TU_{h,i,t}^{care,non-LS}$) in equation (8a), and pecuniary outcomes which feed back into the economy including caregiver worktime losses ($TU_{h,i,t}^{care,LS}$) and effective private labour supplies ($LS_{h,flab,t}$) in equations (8b), (8c), and health unit-costs ($HUC_{i,t}$), household excess health costs ($HC_{h,i,t}$) and decomposed privately ($HCP_{h,i,t}$) and publicly ($HCG_{h,i,t}$) funded excess health costs in equations (8d), (8e), (8f), (8g):

(8a) $$TU_{h,i,t}^{care,non-LS}=\bar{TUrate_{h,i,t}^{care,non-LS}}*\bar{illdur_{i}}*\underset{\begin{array}{c} s\in STRATA\\ g\in G,a\in A \end{array}}{\sum }clin_{h,s,g,a,i,t}^{level},h\in H,i\in \left\{MI-nf,S-nf\right\},\;t\in T$$

(8b) $$TU_{h,i,t}^{care,LS}=\bar{TUrate_{h,i,t}^{care,LS}}*\bar{illdur_{i}}*\underset{\begin{array}{c} s\in STRATA\\ g\in G,a\in A \end{array}}{\sum }clin_{h,s,g,a,i,t}^{level},h\in H,i\in \left\{MI-nf,S-nf\right\},\;t\in T$$

(8c) $$YLD_{h,g,i,t}=\bar{YLDweight_{h,i,t}}*\bar{illdur_{i}}*\underset{\begin{array}{c} a\in A\\ s\in STRATA \end{array}}{\sum }clin_{h,s,g,a,i,t}^{level},h\in H,i\in \left\{MI-nf,S-nf\right\},\;t\in T$$

(8d) $$TU_{h,i,t}^{patient,LS}=\underset{g\in G}{\sum }\bar{partrate_{g}}*YLD_{h,g,i,t},h\in H,i\in \left\{MI-f,S-f\right\},\;t\in T$$

(8e) $$\begin{array}{l} LS_{h,flab,t}=\bar{sklshr_{h,flab}}*(\underset{g\in G}{\sum }\underset{a\in \left\{15-64\right\}}{\sum }\bar{partrate_{g}}*POP_{h,\;g,a,t-1}\\ \;\;\;\;\;\;\;\;\;\;\;\;\;\;\;\;\;\;-\underset{i\in \left\{MI-f,S-f\right\}}{\sum }\left(TU_{h,i,t}^{patient,LS,policy}-TU_{h,i,t}^{patient,LS,count}\right)\\ \;\;\;\;\;\;\;\;\;\;\;\;\;\;\;\;\;\;-\underset{i\in \left\{MI-f,S-f\right\}}{\sum }\left(TU_{h,i,t}^{care,LS,policy}-TU_{h,i,t}^{care,LS,count}\right)),h\in H,flab\in FLAB,\;t\in T \end{array}$$

where $TU_{h,i,t}^{care,LS},TU_{h,i,t}^{care,non-LS}$: Caregiver worktime/leisure time losses; $TU_{h,i,t}^{care,LS,count},TU_{h,i,t}^{care,LS,policy}$: Caregiver worktime losses from counterfactual and policy simulations; $YLD_{h,g,i,t}$: Years Lost due to Disability morbidity; $TU_{h,i,t}^{patient,LS,count},TU_{h,i,t}^{patient,LS,policy}$: Patient worktime losses from counterfactual and policy simulations; $LS_{h,flab,t}$: Household- and labour type-specific effective labour supplies; $illdur_{i}$: Illness duration; $sklshr_{h,flab}$: Household- and labour type-specific labour skill composition shares; FLAB={unskilled,skilled}: Set of unskilled and skilled labour factors.

(8f) $$HUC_{i,t,lag}=\left(\frac{GDPDEF_{t}}{\bar{GDPDEF_{t_{0}}}}\right)*\bar{HUC_{i,t_{0},lag}},h\in H,flab\in FLAB,\;t\in T,lag\in LAG$$

(8g) $$HC_{h,i,t}=\overset{3}{\underset{lag=0}{\sum }}HUC_{i,t,lag}*\underset{\begin{array}{c} s\in STRATA\\ g\in G,a\in A \end{array}}{\sum }clin_{h,s,g,a,i,t-lag}^{level},h\in H,i\in \left\{MI-nf,S-nf\right\},\;t\in T$$

where $HUC_{i,t,lag}$: Lagged illness-specific health unit-costs; $HC_{h,i,t}$: Public funded illness-specific excess health costs; $GDPDEF_{t}$: GDP deflator; LAG  = {0,1,2,3}: Illness-specific lag structure for formal health costs. The module assumes, in equation (8d), that YLD morbidity impacts approximate illness-specific patient time losses, and that they, when corrected for labour force participation rates approximate patient worktime losses. The module also assumes, in equation (8f), that health unit costs increases, over time, in line with the GDP deflator price index. Finally, equation (8g) specifies that public funded formal health costs accumulates over the lag-time period lag∈LAG={0,1,2,3} where health unit costs for lags time 1–3 are only non-zero for non-fatal stroke since average illness duration for non-fatal MI is 28 days (WHO, 2013).

Parametrization of the caregiver leisure and worktime time loss equations, equations (8a), (8b), were based on Thai-specific average time loss estimates (Riewpaiboon, Riewpaiboon, Ponssongnern, & van den Berg, 2009),^2^ while parametrization of the YLD and labour supply equations, equations (8c), (8d), (8e), were based YLD weights from the literature (WHO, 2013) and Thai-specific skill-shares and workforce participation rates (NSO 2014). Initial values of Thai-specific hospital unit costs ($\bar{HUC_{i,t_{0}}}$), in equation (8f), were also derived from the literature, including MI-related hospital unit costs (Anukoolsawat, Sritara, & Teerawattananon, 2006) and stroke-related hospital unit costs (Khiaocharoen, Pannarunothai, & Zungsontiporn, 2012).

### 2.7 Land use change module

Finally, we employ a simplified equilibrium-type environmental LUC satellite module to measure LUC-related GHG emissions in units of mega-tonnes (Mt) of CO_2_-equivalents (CO_2_-eq). We specify our environmental module to focus, narrowly, on measurement of direct LUC impacts on carbon sequestration (Jensen et al., 2019). Our CGE model simulates regional land use over a detailed set of agricultural production sectors, including six primary food crops (which aggregates to our primary food crop sector in Table 1) and one primary non-food crop.^3^ Specifically, our model makes the simplifying equilibrium assumption that crop-specific LUC change occurs proportionally between sectors experiencing LUC losses and sectors experiencing LUC gains in equations (10a), (10b), (10c). The modelling of agricultural activity-specific land factor demand ($FLAND_{act,land,t}$) in the CGE model, based on Constant Elasticity of Substitution (CES) production functions and governed by first order conditions for profit maximization in equation (9), then allows us to measure changes in LUC-related GHG emissions ($GHG_{t}$) in equation (10d):

(9) FLANDact,flnd,t=(δact,flndVA¯αactVA¯ρactVA¯)1(1+ρactVA¯)∗(PVAact,tWFLANDact,flnd,t)1(1+ρactVA¯)∗QVAact,t,act∈ACTagr,flnd∈FLND,t∈T

(10a) $$\Delta FLAND_{flnd,t}^{+}=\underset{act\in ACTagric}{\sum }\left(1\left[\Delta FLAND_{act,flnd,t}>0\right]*|\Delta FLAND_{act,flnd,t}|\right),flnd\in FLND,\;t\in T$$

(10b) $$\Delta FLANDshr_{act,flnd,t}^{+}=\left(\frac{1\left[\Delta FLAND_{act,flnd,t}>0\right]*|\Delta FLAND_{act,flnd,t}|}{\Delta FLAND_{flnd,t}^{+}}\right),\;act\in ACTagric,flnd\in FLND,t\in T$$

$$\Delta FLANDshr_{act,flnd,t}^{-}=\left(\frac{1\left[\Delta FLAND_{act,flnd,t}\leq 0\right]*|\Delta FLAND_{act,flnd,t}|}{\Delta FLAND_{flnd,t}^{+}}\right),\;act\in ACTagric,flnd\in FLND,t\in T$$

(10c) $$\Delta FLAND_{act1,act2,flnd,t}^{transit-specific}=\Delta FLANDshr_{act1,flnd,t}^{-}*\Delta FLANDshr_{act2,flnd,t}^{+}*\Delta FLAND_{flnd,t}^{+},$$

act1,act2∈ACTagric,flnd∈FLND,t∈T

(10d) $$\Delta GHG_{t}=\underset{\begin{array}{c} act1\in ACT\\ act2\in ACT\\ flnd\in FLND \end{array}}{\sum }EmissCoef_{act1,act2,t}*\Delta FLAND_{act1,act2,flnd,t}^{transit-specific},\;t\in T$$

where $WFLAND_{act,flnd,t},FLAND_{act,flnd,t}$: activity-specific land return and land demand; $PVA_{act,t},QVA_{act,t}$: activity-specific value added price and value added production; $\Delta FLAND_{flnd,t}^{+},\Delta FLAND_{flnd,t}^{-}$: sums of positive/negative land use changes (ha); $\Delta FLANDshr_{act,flnd,t}^{+}/\Delta FLANDshr_{act,flnd,t}^{-}$: activity-specific shares of positive/negative land use changes (%); $\Delta FLAND_{act1,act2,flnd,t}^{transit-specific}$: crop transition-related land use changes (ha), positive signs indicating changes from activity1→activity2 crop production; $\Delta GHG_{t}$: Change in greenhouse gas (GHG) emissions from agricultural crop transition-related carbon sequestration (Mt CO2-eq); ($\bar{\alpha_{act}^{VA}},\bar{\delta_{act,flnd}^{VA}},\bar{\rho_{act}^{VA}}$): activity-specific CES value added production function parameters; $EmissCoef_{act1,act2,t}$: plot- and activity-specific GHG emission coefficients (Mt CO2-eq/ha), emissions change from activity1→activity2 crop production change; $FLND=\left\{land^{central\&cast},land^{north},land^{northeast},land^{south}\right\}$: Set of central & eastern, northern, north-eastern, and southern region land factors. As mentioned, above, in the CGE model subsection, standard elasticity values ($\sigma_{act}^{VA}=\frac{1}{1+\bar{\rho_{act}^{VA}}}=0.6$) were used for our CES factor input demand specifications in equation (9), allowing for standard calibration of the remaining value added production function parameters ($\bar{\alpha_{act}^{VA}},\bar{\delta_{act,flnd}^{VA}}$). Finally, Thai-specific LUC emission coefficients (Silalertruksa & Gheewala, 2012) were used to parametrize the GHG emissions equation (10d).

### 2.8 Policy indicators

As outlined above, our multi-sector and multi-dimensional dynamically-recursive MED-health model framework produces a range of nutritional, health, demographic, economic and environmental indicators over our 20 year time horizon 2016–2035. In what follows, we focus on a few core indicators: nutritional indicators include average long-run SFA, MUFA and PUFA energy intake shares ($\frac{1}{\left|H\right|}\underset{h\in H}{\sum }e_{h,T}^{SFA},\frac{1}{\left|H\right|}\underset{h\in H}{\sum }e_{h,T}^{PUFA},\frac{1}{\left|H\right|}\underset{h\in H}{\sum }e_{h,T}^{MUFA}$) and average long-run Total:HDL cholesterol biomarkers ($\frac{1}{\left|H\right|}\underset{h\in H}{\sum }chol_{h,T}$); for health, we present cumulative incident cases from MI and stroke $\left(\underset{t\in T}{\sum }\underset{g\in G,a\in A}{\sum }clin_{h,\;g,a,i,t}^{level},h\in H,i\in \left\{MI-nf,Stroke-nf\right\}\right)$ and premature deaths from MI and stroke $\left(\underset{t\in T}{\sum }\underset{g\in G,a\in A}{\sum }clin_{h,\;g,a,i,t}^{level},h\in H,i\in \left\{MI-f,Stroke-f\right\}\right)$ our demographic outcomes include cumulative population ($\underset{t\in T}{\sum }\underset{h\in H,g\in G,a\in A}{\sum }POP_{h,g,a,t}$) and workforce ($\underset{t\in T}{\sum }\underset{h\in H,flab\in FLAB}{\sum }LS_{h,flab,t}$) impacts; economic outcomes are measured in terms of cumulative real GDP ($\underset{t\in T}{\sum }GDP_{t}^{real}$) impacts; and environmental impacts are measured in terms of long-run GHG emissions of CO_2_-Eq ($GHG_{T}$).

Real GDP impacts are measured in two different ways. The standard method for CGE analysis allows real private consumption to vary and this method is employed to determine overall policy impacts. However, in order to enable further analysis and decomposition of tariff impacts, we also perform efficiency simulations where total real private consumption is fixed at the counterfactual growth path (substitution between household-specific consumption items is still allowed). The efficiency simulations are not used to measure non-economic outcomes, but are simply used to isolate fiscal tax efficiency impacts on the production/investment side of the economy and thereby allow for clean measurement of potential inefficiencies of tariff instruments. Isolation of real GDP efficiency impacts on the production/investment side is ensured since real government consumption is fixed at the counterfactual growth path, and since the real trade balance (real exports – real imports) is fixed as part of the external model closure (where the fixed Balance of Payments is cleared by a flexible exchange rate). In addition, marginal real GDP health pathway impacts of a given tariff simulation are valued (and reported) based on simulation of health impacts (workforce ($LS_{h,flab,t}^{policy}-LS_{h,flab,t}^{count}$) and formal health cost ($HC_{h,i,t}^{policy}-HC_{h,i,t}^{count}$) impacts) without imposing the underlying tariff instrument.

Estimates of policy impacts are produced by comparing the results of a policy simulation with a counterfactual solution of the model. In our scenarios, discussed below, the counterfactual is represented by either business as usual or an alternative policy scenario for comparison with the policy simulation.

### 2.9 Scenarios and model closure

**Fig. 2:**
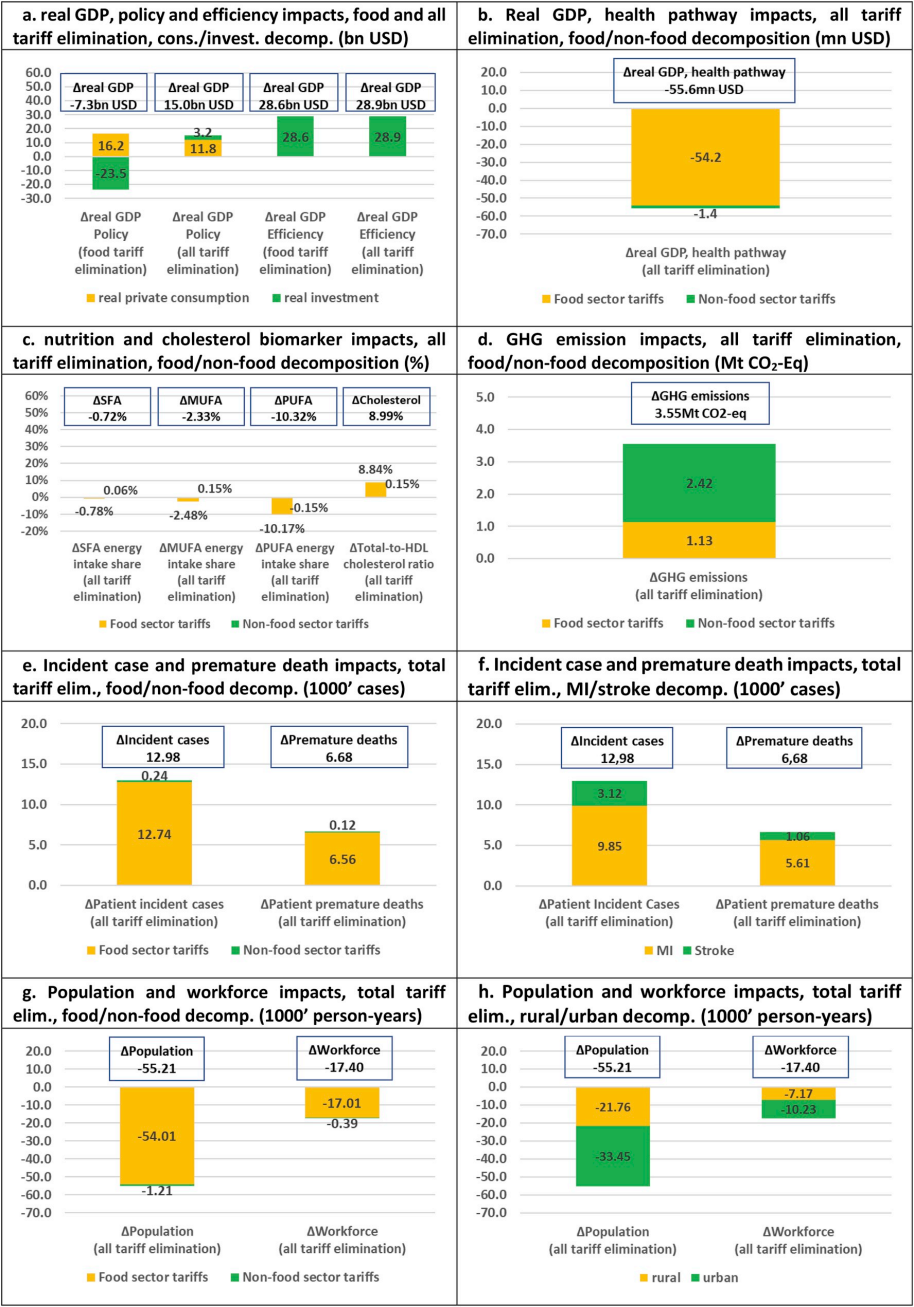
**Elimination of existing import tariffs (absolute impacts and decompositions)**.

**Fig. 3:**
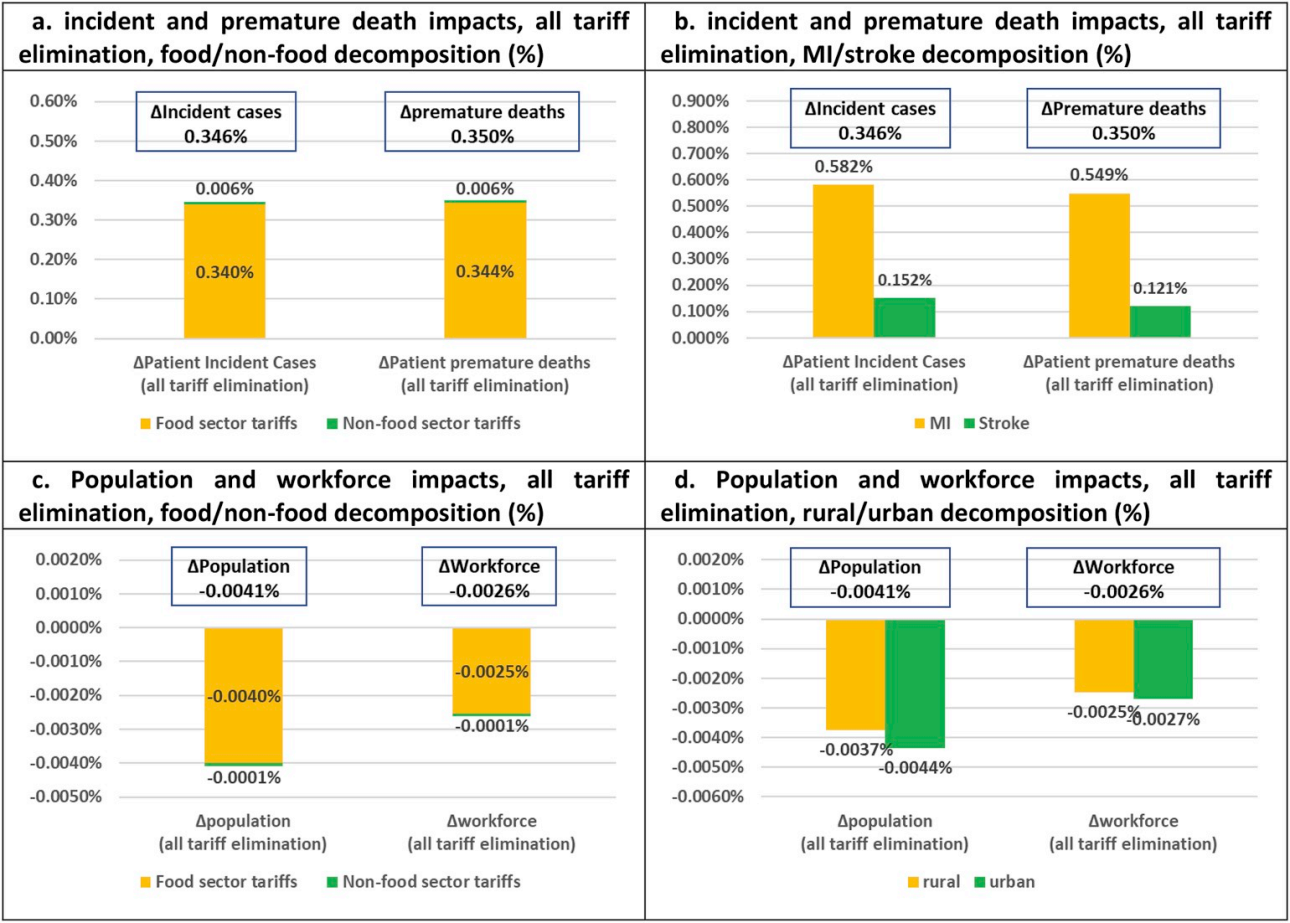
**Elimination of all existing import tariffs (relative impacts and decompositions)**.

**Fig. 4:**
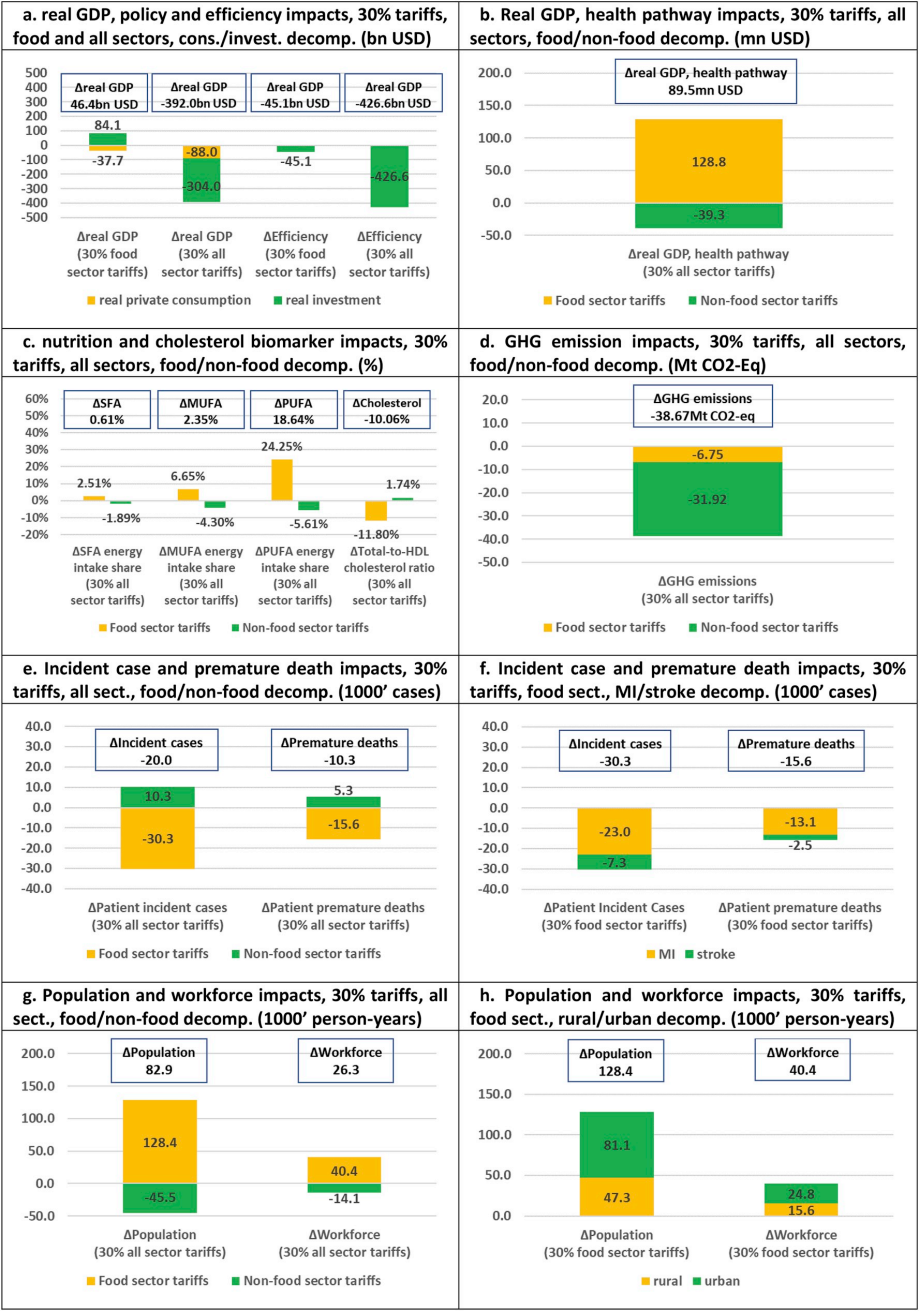
**Uniform 30% food sector and 30% all sector import tariffs (absolute impacts and decompositions)**. Note: Counterfactual simulation has eliminated all import tariffs.

**Fig. 5:**
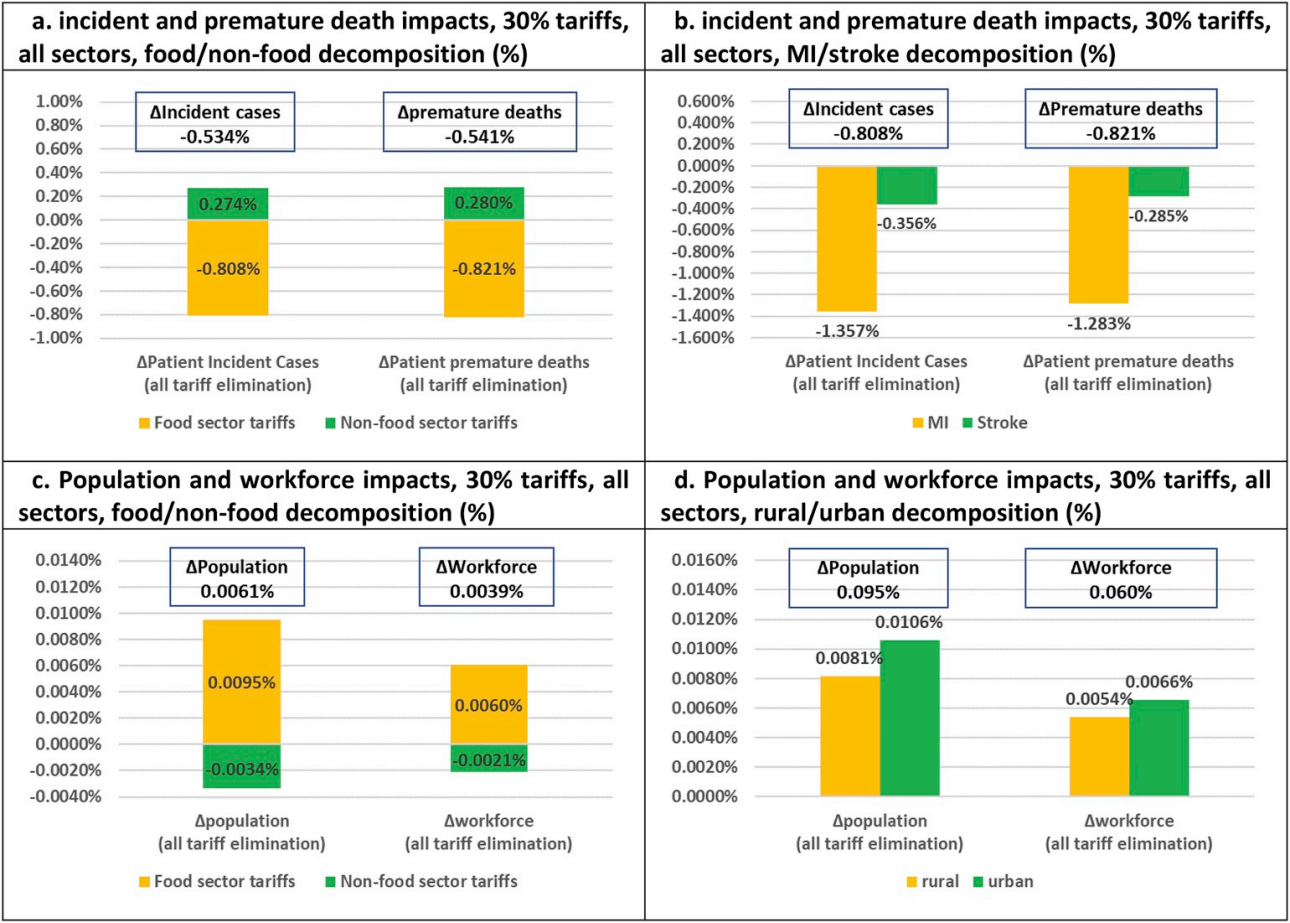
**Uniform 30% import tariffs on all sectors (relative impacts and decompositions)**. Note: Counterfactual simulation has eliminated all import tariffs.

**Fig. 6:**
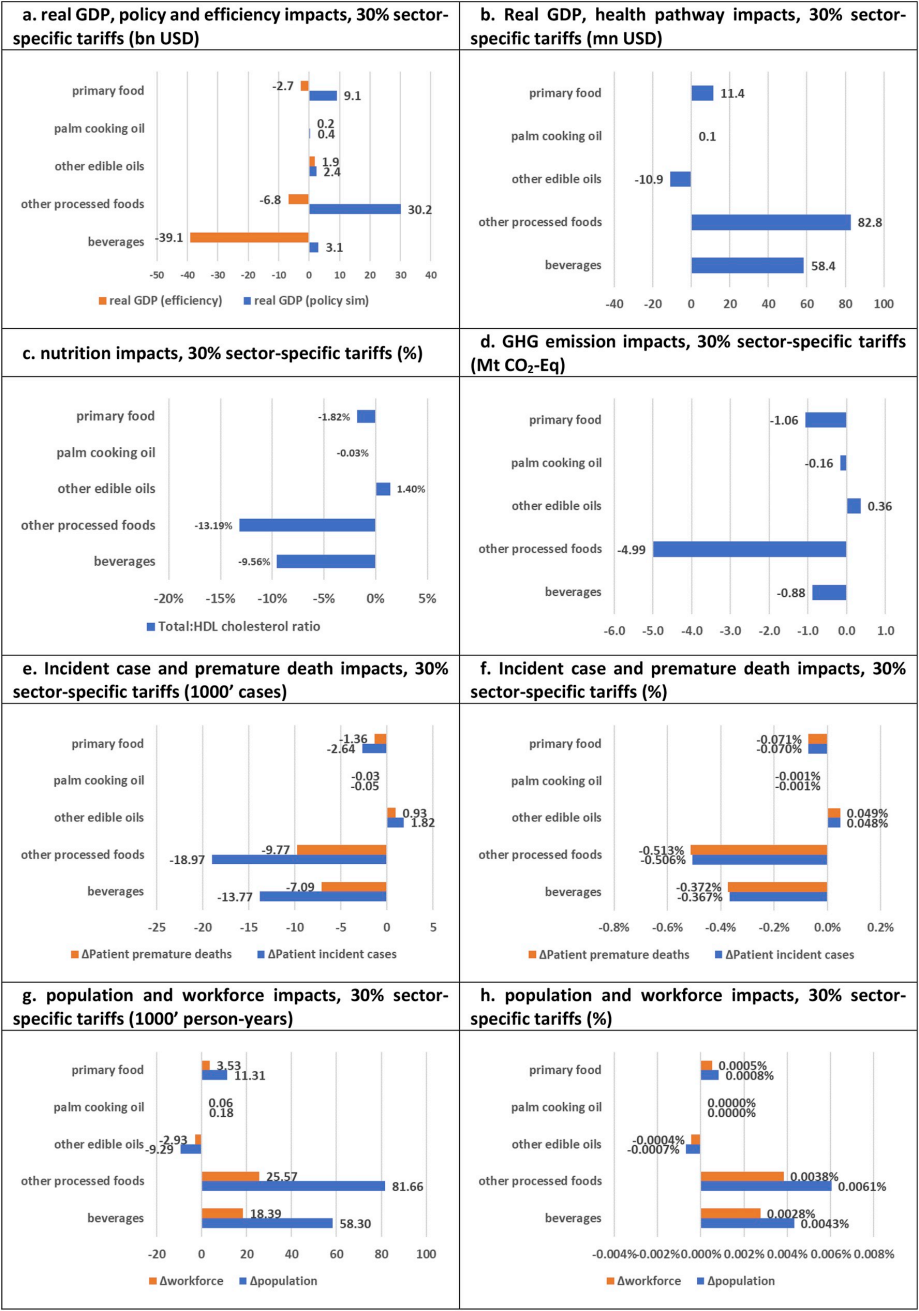
**Sector-specific 30% food import tariffs (relative impacts)**. Note: Counterfactual simulation has eliminated all import tariffs.

We analyse three sets of policy scenarios including one set of aggregate scenarios, measured relative to a ‘business as usual’ (BaU) counterfactual, and two sets of aggregate and sector-specific scenarios, which are measured relative to a ‘no import tariff distortion’ (NITD) counterfactual where all import tariffs have been eliminated. The first set of aggregate scenarios, which are measured against the BaU counterfactual and used to assess the existing import tariff schedule, involves three simulations including (1) elimination of all existing food import tariffs, (2) elimination of all existing non-food import tariffs, and (3) elimination of all existing food and non-food import tariffs combined (results presented in Fig. 2, Fig. 3). The second set of aggregate scenarios, measured against the NITD counterfactual, is used to assess stylized tariff increases based on two simulations including (1) imposition of 30% uniform import tariffs across all food sectors, and (2) imposition of 30% uniform import tariffs across all food and non-food sectors (results presented in Fig. 4, Fig. 5). Finally, the third set of disaggregate sector-specific scenarios, measured against the NITD counterfactual, involves imposition of 30% sector-specific food import tariffs for each of our five food sectors individually. The latter set of scenarios allows us to decompose the aggregate impact of imposing a 30% import tariff across all food sectors at the same time (results presented in Fig. 6).

Our stylized 30% tariff rate on food and non-food commodities was chosen because it encompasses all current tariff rates including the beverage sector with a 22.7% tariff rate (Table 1) and since most Thai-specific WTO maximum bound duties are ≥30% (several major sectors, including clothing and machinery, have maximum bound duties = 30% while all primary and processed food sectors have maximum bound tariffs ≥50%) (WTO, 2019). An additional motivation was to ensure that our food policy import tariffs would be effective. The fiscal food policy literature dictates that domestic food policy taxes should be set above a 15% minimum threshold for effectiveness (Niebylski, Redburn, Duhaney, & Campbell, 2015). Accounting for the fact that there may be potentially limited price feed-through from import tariffs to domestic prices, we set our stylized import tariff rates at 30% in order to ensure that they would be effective as food policy tax instruments.

In the following, all scenarios are simulated with a standard neoclassical model closure, where prices clear all domestic markets, a flexible real exchange rate clears the (fixed) current account of the balance of payments, and real government consumption is fixed at the counterfactual growth path. Our three scenarios are analysed consecutively in the following three sub-sections.

## 3 Results

### 3.1 Elimination of existing import tariff structure

The results of eliminating all existing food and non-food import tariffs are presented in Fig. 2, Fig. 3. The cumulative real GDP impacts of food tariff elimination include a USD -7.3bn policy impact and a USD 28.6bn efficiency impact over our 20 year time horizon (Fig. 2a). Hence, while simple tariff elimination may reduce cumulative real GDP (tariff elimination reduces the purchase price of imported goods, and this increases consumption and reduces savings/investment and thereby reduces GDP in the longer term), the results show that potentially large economic efficiency (and long-term welfare) gains can be reaped by eliminating food import tariffs in Thailand. Interestingly, efficiency gains from eliminating all tariffs are only marginally higher (USD 28.9bn), indicating that the main distortions from the current tariff structure derives from tariffs on primary and secondary food sectors.

While full Thai trade liberalization would bring economic efficiency gains, our nutritional, health, and environmental indicators would be adversely affected. SFA, MUFA and PUFA energy intake shares decline across the board. The −0.7% reduction in SFA intake shares is, in principle, beneficial, but due to much larger MUFA and PUFA intake share reductions of −2.3% and −10.3%, the average cholesterol biomarker is driven up by 9.0% (Fig. 2c). This, in turn, drives up CVD clinical outcomes by respectively 12,980 incident cases and 6680 premature deaths over our 20 year time horizon (Fig. 2f). Demographic ripple effects include a cumulative population reduction of 55,210 person-years (Fig. 2g), or 4.1 persons per 100,000 population (Fig. 3c). Hence, while existing import tariffs marginally increase Thai SFA intake shares, the tariff structure unwittingly protects against CVD illness in Thailand.

Our disaggregated results indicate that the current tariff structure has a particularly positive impact on containing MI in Thailand. Full tariff elimination would increase MI incident cases and deaths by respectively 0.58% and 0.55%, while stroke cases and deaths would increase by respectively 0.15% and 0.12% (Fig. 3b). In absolute terms, clinical outcomes would increase by respectively 9850 cases/5610 deaths and 3120 cases/1060 deaths over our 20 year time horizon (Fig. 2f). The existing tariff structure also turns out to have a slightly positive urban health bias. Hence, full elimination of tariffs would reduce population and workforce numbers in urban areas by 4.4 and 2.7 persons per 100,000 population/workers, and in rural areas by 3.7 and 2.5 persons per 100,000 population/workers. Finally, tariff elimination would reallocate primary food production towards sectors with reduced carbon sequestration potential and raise LUC-related GHG emissions by 3.55 Mt CO_2_-Eq (Fig. 2d). Hence, in addition to protecting against Thai unhealthy eating and CVD-related (urban MI) disease burdens, the current tariff structure protects against environmental damage.

The economic impact of the health pathway, including labour market and formal healthcare costs, is small compared to the broader distortionary effects of the tariff structure. The real GDP health pathway impact of total tariff elimination is USD -55.6mn (Fig. 2b) or −0.2% of the overall efficiency impact (USD 28.9bn). While it is interesting to note that the health economic impact of food tariff elimination, alone, is USD -54.2mn, and that food tariffs dominate economic health impacts, it is also clear that economic efficiency considerations cannot justify maintaining the existing protective tariff structure. Thai policy makers, who consider liberalizing (food) tariffs, would therefore be facing a trade-off between economic efficiency on the one hand, and increased unhealthy eating, worsening clinical health and demographic outcomes, and increased environmental damage on the other. In other words, a continued Thai drive towards tariff liberalization and economic efficiency may come at the expense of reduced health and environmental sustainability of food consumption and production.

### 3.2 Imposition of uniform 30% import tariffs

In this section, we study the broader health, economic, and environmental impacts of tariff protection through imposition of 30% aggregate food import tariffs and 30% aggregate import tariffs across all sectors (where the counterfactual involves no import tariff distortions). The results are presented in Fig. 4, Fig. 5. While the food tariff policy simulation has a positive cumulative USD 46.4bn real GDP impact (a tariff-induced USD -37.7bn private consumption reduction leads to increased cumulative private savings and a USD 84.1bn investment crowding-in), the efficiency simulation impact is USD -45.1bn over our 20 year time horizon (Fig. 4a). This confirms that uniform food sector tariffs are strongly distortionary and not economic welfare enhancing for Thailand. Not surprisingly, tariffs on the dominant non-food sector are significantly more distortionary. Hence, a uniform 30% tariff across all food and non-food sectors reverses the USD 45.1bn food policy gain and reduce cumulative real GDP by USD 392.0bn, while the negative efficiency impact is increased by an order of magnitude to USD -426.6bn over our 20 year time horizon. Neither uniform food or non-food import tariffs are welfare enhancing for Thailand.

Our stylized 30% tariff simulations confirm our findings from existing tariff simulations: while uniform protective tariffs lead to adverse economic impacts, our nutritional, health, and environmental indicators generally improve across the board. In general, food tariffs increase fatty acid intake shares while they are reduced by non-food tariffs. Nutritional impacts are, however, not surprisingly driven by the beneficial impacts of food tariffs. Looking specifically at 30% food tariffs, we note that an adverse nutritional increase in the SFA energy intake share (2.5%) is dominated by a particularly strong 24.3% increase in the PUFA intake share. The food tariff-specific PUFA impact is the main driving force behind the −11.8% cholesterol biomarker reduction in the food tariff simulation, and the −10.1% reduction in the combined food and non-food tariffs simulation (Fig. 4c). Hence, despite relatively modest import shares for primary and processed food sectors (Table 1), food tariffs are more potent than non-food tariffs in affecting nutritional outcomes. Our fatty acid composition results suggest that WHO guidelines and policy indicators to address unhealthy eating (WHO, 2017b) could require some nuance, since it is not necessarily the reduction in SFA energy intake shares but the composition of nutritional fatty acid intakes which matters for reducing cholesterol-related CVD clinical outcomes.

Our results confirm that beneficial and adverse nutritional impacts of respectively food and non-food tariffs drive clinical health outcomes. Uniform 30% food tariffs reduce CVD outcomes by 30,300 incident cases and 15,600 deaths (Fig. 4e) and illness-specific disease burdens by more than 0.8% (Fig. 5b), while uniform 30% non-food tariffs adversely increase CVD outcomes by 10,300 incident cases and 5300 deaths (Fig. 4e) and illness-specific disease burdens by almost 0.3% over our 20 year time horizon (Fig. 5b). A strategy of trade protection, with the aim of limiting Thai CVD clinical health outcomes, would therefore, naturally, have a focus on trade protection of primary and processed food sectors. The same picture emerges from the demographic impacts. While cumulative population and workforce indicators expand by 128,400 and 40,400 person-years in the food tariff scenario, they decline by 45,500 and 14,100 person-years when tariffs are limited to non-food sectors (Fig. 4g). Uniform 30% food tariffs would save 9.5 persons per 100,000 population and 6.0 workers per 100,000 workforce (Fig. 5d). Interestingly, environmental impacts are beneficial regardless of whether 30% food tariffs (−31.9 Mt CO_2_-Eq) or 30% non-food tariffs (−6.8 Mt CO_2_-Eq) are imposed, but food tariffs are again more potent.

In summary, uniform food tariffs tend to improve nutritional, clinical health, demographic, and environmental indicators, while uniform non-food tariffs tend to do the reverse (except for environmental outcomes). This is also evident in the economic health pathway impacts where uniform 30% food tariffs raise cumulative real GDP by USD 128.8mn while uniform 30% non-food tariffs reduce real GDP by USD 39.3mn (Fig. 4b). However, similar to the existing tariff simulations, analysed above, the real GDP health pathway impact of uniform 30% food tariffs (USD 128.8mn) is dwarfed by the efficiency impact (USD -45.1bn). Hence, the imposition of protective food import tariffs to incentivize healthy eating cannot be argued on economic efficiency grounds, but needs to focus on their potential cost-effectiveness as health interventions (see discussion below).

### 3.3 Imposition of 30% food sector-specific import tariffs

Finally, we study the health, economic, and environmental impacts of food sector-specific tariff protection through imposition of 30% sector-specific food tariffs (where the counterfactual again involves no import tariff distortions). Results are presented in Fig. 6. While real GDP policy gains from protection of “other processed foods” sectors (USD 30.2bn, Fig. 6a) account for two-thirds of total food policy gains (USD 46.4bn, Fig. 4a), efficiency losses from protection of “beverages” (USD -39.1bn, Fig. 6a) account for almost 90% of total efficiency losses (USD -45.1bn, Fig. 4a). Hence, fiscal food policy distortions would mainly arise from imposition of import tariffs on the relatively large Thai beverages sector.

Despite the general inefficiency of imposing fiscal food policy tariffs, it is interesting to note that Thailand is characterized by a second-best environment where efficiency gains can be reaped from increasing import tariffs on the “palm cooking oil” and “other edible oils” sectors. However, due to small import shares, the health gains from imposing “palm cooking oil” tariffs is limited (in the order of 50 incident cases/30 premature deaths avoided or 0.001% burden reductions, Fig. 6e–f). Furthermore, due to adverse substitution patterns towards unhealthy palm cooking oil consumption, a 30% tariff-induced reduction in “other edible oil” imports (mainly reduced soybean oil imports) leads to adverse CVD health outcomes (1820 new incident cases/930 new deaths; 0.048–0.049% burden increases, Fig. 6e–f). Despite the increased economic efficiency, import tariffs on edible oil sectors are therefore not potent (palm cooking oil) or warranted (other edible oils).

The most potent fiscal food policy tariffs, in terms of nutritional, health and demographic impacts, are tariffs on “beverages” (13,770 incident cases avoided/7090 deaths avoided/0.37% burden reductions, Fig. 6e–f) and “other processed foods” (18,970 incident cases avoided/9770 deaths avoided/0.51% burden reductions, Fig. 6e–f). Specifically, sector-specific 30% tariffs would reduce cholesterol biomarkers by 9.6% (“beverages”) and 13.2% (“other processed foods”) (Fig. 6c), and thereby improve demographic population outcomes by 58,300 person-years/4.3 persons per 100,000 population (beverages) and 81,660 person-years/6.1 persons per 100,000 population (other processed foods) (Fig. 6g–h). The positive health outcomes are accompanied by beneficial sector-specific GHG emission reductions amounting to 0.9 Mt CO_2_-Eq (“beverages”) and 5.0 Mt CO_2_-Eq (“other processed foods”) (Fig. 6d), and by positive sector-specific real GDP health pathway impacts of USD 58.4mn (“beverages”) and USD 82.8mn (“other processed foods”) (Fig. 6b). However, despite the relative potency of the fiscal food policy tariffs instruments for these sectors, the health pathway impacts are again dwarfed by real GDP efficiency losses of respectively USD 39.1bn and USD 6.8bn (Fig. 6a). This reaffirms the conclusion that protective food import tariffs to incentivize healthy eating cannot be argued for on economic efficiency grounds. Instead, adoption would need to rest on their potential cost-effectiveness as health interventions (see discussion below).

## 4 Discussion and conclusions

The results of eliminating existing food and non-food import tariffs indicate that the main distortions from the current tariff structure derive from tariffs on primary and secondary food sectors, and in particular, from high tariffs on the large beverages food sector. Overall, our tariff elimination results suggest that existing (food) tariffs protect against cholesterol-related CVD illness in Thailand by increasing energy intake shares of unsaturated fatty acids and reducing the Total-HDL cholesterol biomarker. Altogether, the tariff structure reduces MI/stroke burdens by 0.55–0.58%/0.12–0.15% and saves 55,210 person-years over our 20 year time horizon. The tariff structure also has a slightly pro-urban health protection bias saving 4.4 persons per 100,000 population in urban areas and 3.7 persons 100,000 population in rural areas. At the same time, existing tariffs cause an economic efficiency loss of USD 28.6 bn indicating a cost of ≈USD 500,000 per person-year saved. This exceeds standard cost-effectiveness thresholds of developed countries (typically around USD 30–35,000 per person-year) by an order of magnitude, and thresholds of developing nations by even more. Non-pecuniary valuation of long run GHG emission reduction benefits according to World Bank (WB) Guidelines (WB, 2017) would not change our conclusions. The suggested valuation of GHG emissions at USD 50–100 per tonne of CO_2_-eq (ibid.) would only value the 3.55 Mt CO_2_-eq emission reduction at < USD 360mn.

In summary, while the existing tariff structure can be motivated by pointing to beneficial nutritional, clinical health, demographic, and environmental impacts, the tariff structure causes large economic efficiency losses and can, therefore, not be considered cost-effective as a public health intervention against CVD illness in Thailand. Nonetheless, Thai policy makers, who consider liberalizing (food) tariffs, do face a trade-off between economic efficiency on the one hand, and increased unhealthy eating, worsening clinical health and demographic outcomes, and increased environmental damage on the other. A continued Thai drive towards tariff liberalization and economic efficiency should therefore carefully consider that this may come at the expense of reduced health and environmental sustainability of food consumption and production.

Our stylized 30% tariffs demonstrated that food and non-food tariffs alike cause economic efficiency losses on the one hand and reduced environmental LUC-related GHG emissions on the other. Without considering health impacts, policy makers therefore face an economic-environmental trade-off when considering imposition of protective tariffs. In terms of nutrition and clinical health, food tariffs generally improved nutritional, health and demographic indicators, while non-food tariffs did the opposite: uniform 30% food tariffs reduced CVD outcomes by 30,300 incident cases/15,600 deaths and illness-specific burdens by more than 0.8%, while uniform 30% non-food tariffs, on the other hand, increased CVD outcomes by 10,300 incident cases/5300 deaths and illness-specific disease burdens by almost 0.3%. Focusing on food sectors, imposition of uniform 30% tariffs would save 128,400 person-years or 9.5 persons per 100,000 population, but would be accompanied by a USD 45.1bn economic efficiency loss. Again, the implied cost (of ≈USD 350,000 per person-year saved) exceeds standard cost-effectiveness thresholds of developed and developing countries by (more than) an order of magnitude, and non-pecuniary valuation of long run GHG emission reduction benefits of 31.9 Mt CO_2_-Eq (<USD 3.2bn) would not change this conclusion.

Interestingly, while nutritional cholesterol impacts of uniform food tariffs are generally found to be beneficial due to increased unsaturated fatty acid energy intake shares, uniform 30% food tariffs were also found to cause a marginal 2.5% increase in SFA energy intake shares. This result suggests that WHO guidelines, and their SFA-focussed policy indicators to address unhealthy eating (WHO, 2017b), could require some nuance, since it is not necessarily the reduction in SFA energy intake shares but the composition of fatty acid intakes which matters for reducing cholesterol-related CVD clinical outcomes. At the same time, our Thailand-specific results confirm previous non-Thai results, which indicate that increased import shares of food commodities, both in terms of expenditures and caloric intakes, tends to be correlated with unhealthy eating and adverse health outcomes (obesity), among the population of the importing country (Estimé et al., 2014).

In terms of individual food sector interventions, it is interesting to note that Thailand seems to be characterized by a second-best environment where economic efficiency gains can be reaped from increasing import tariffs on edible oils. Nonetheless, fiscal food policy tariffs on edible oils are poor public health instruments in Thailand: tariffs on palm cooking oil are not potent due to small import shares, and imposing tariffs on other edible oils (mainly soybean oil) would lead to adverse nutritional, clinical health and demographic outcomes due to substitution towards unhealthy palm cooking oil consumption.

The majority of food tariff-related economic distortions stem from imposing tariffs on the beverages sector. While a 30% tariff on the beverages sector was found to reduce CVD clinical outcomes by 13,770 incident cases and 7090 deaths (0.37% reduction in burdens) and save 58,300 person-years, it also leads to a USD 39.1bn efficiency loss. The implied cost (of ≈USD 670,000 per person-year saved) again exceeds standard cost-effectiveness thresholds of developed and developing countries by more than an order of magnitude, and non-pecuniary valuation of long run GHG emission reduction benefits of 0.9 Mt CO_2_-Eq (<USD 100mn) does not change our conclusion that a general tariff on the beverages sector would represent a cost-ineffective public health intervention to control cholesterol-related CVD in Thailand.

In terms of the other processed foods sector, a 30% tariff, while reducing CVD clinical outcomes by 18,970 incident cases and 9770 deaths (0.51% reduction in burdens) and saving 81,660 person-years (6.1 persons per 100,000 population), would also lead to a USD 6.8bn efficiency loss. The implied cost of ≈USD 84,000 per person-year saved is about three times as high as standard cost-effectiveness thresholds of developed countries, and non-pecuniary valuation of long run GHG emission reduction benefits of 5.0 Mt CO_2_-Eq (<USD 500mn) would only reduce the cost to ≈ USD 78,000 per person-year saved.

In summary, we find that uniform tariffs on food and non-food sectors as well as sector-specific tariffs on individual edible oil sectors and broader beverages and processed foods sectors (other than edible oils) do not represent cost-effective public health interventions to control cholesterol-related CVD in Thailand. Nonetheless, fiscal food policy tariffs generally tend to improve both nutritional, clinical health, demographic, and environmental indicators, indicating that policy makers from Thailand and abroad, including WHO, would do well in considering food sector tariffs as a potential intervention to maintain combined health and environmental sustainability of food consumption and production systems.

Several caveats can be noted to motivate continued exploration of food import tariffs as a public health instrument. First, we have only analysed broad food sectors; second, we have only analysed the compositional impact of fatty acid intakes on cholesterol-related CVD outcomes; and third, our conservative back-of-the-envelope cost-effectiveness measures do not account for likely complementary improvements in disability-adjusted life years. Specifically, we have not disaggregated the large beverages sector between Sugar-Sweetened Beverages (SSB) and other beverages. SSB consumption is well-known for causing obesity and overweight, which are well-known risk factors for CVD clinical outcomes (beyond compositional fatty acid intake effects). Our analysis of a general beverages sector tariff is not fully representative of a specific SSB sector tariff. A full SSB-specific analysis of the usefulness of our food policy tariff instrument as a public health instrument would require further disaggregation of the beverages sector and additional analyses of the importance of nutritional intake levels and obesity impacts (beyond our nutritional composition focus). That being said, the very high economic efficiency losses and accompanying health policy cost of applying general beverages sector tariffs (≈USD 660,000–670,000 per person-year saved) does suggest that SSB-specific import tariffs are unlikely to be cost-effective in Thailand.

A more promising area for exploring food import tariffs as a public health instrument in Thailand is the processed foods sector (excluding edible oils). This sector covers a number of nutritionally diverse food groups including cereal grain products, prepared and preserved fish or vegetables, fruit juices and vegetable juices, prepared and preserved fruits and nuts, cereal flours, meal and pellets, sugars and sugar syrups, bakery products, chocolate and sugar confectionery, etc. The relatively moderate average public health cost of applying general processed food sector tariffs (≈USD 78,000–84,000 per person-year saved) suggests that individual food group import tariffs could well be cost-effective as a public health instrument to control cholesterol-related CVD illness in Thailand. This is especially so considering the very conservative nature of our simulation results, which does not account for obesity-related changes in energy intake levels, and the conservative nature of our cost-effectiveness measures which do not account for likely complementary improvements in disability-adjusted life years.

Finally, we note that our 30% stylized import tariffs, generally, are feasible as trade policy instruments and/or health policy interventions, since WTO bound tariffs ≥30% for all food sectors and most non-food sectors. An important caveat to our conclusions regarding employing fiscal food tariffs as a public health instrument applies to the edible oil sector. While we demonstrate that palm cooking oil import tariffs lack potency due to low import shares, the import shares, themselves, are kept low due to non-tariff barriers (NTBs) including variable import and export quotas. Thailand enforces NTBs in order to protect domestic production of palm cooking oil for domestic production. However, since NTBs are notoriously difficult to quantify, it was not possible to infer the public health cost of these NTBs. Due to low trade shares, the removal of NTBs is unlikely to be a more potent intervention, than the (impotent) palm cooking oil tariffs, for affecting CVD illness in Thailand. This is, however, an empirical matter and we leave it for future research to assess this conjecture.

In conclusion, the existing import tariff structure protects against cholesterol-related CVD illness in Thailand and lowers agricultural LUC-related GHG emissions, but at a cost of ≈USD 500,000 per person-year saved, the tariff structure is not, by itself, cost-effective as a public health policy instrument. This conclusion is not likely to change even if adjustment was made for additional obesity and overweight-related health impacts and morbidity-related improvements in disability-adjusted life years. Since the imputed economic value of environmental co-benefits is relatively small as well, the inefficient Thai tariff structure must be motivated by other considerations, e.g. food security or infant industry protection.

Surprisingly, our stylized 30% tariff simulations further indicate that food and non-food sector import tariffs, across the board, are cost-ineffective as public health interventions - the least costly “other processed foods” import tariff intervention carry a cost of 78–84,000 USD per person-year saved. However, due to the broadly defined nature of the “other processed foods” food group and the conservative nature of our cost-effectiveness calculations, we conjecture that individual food group import tariffs could well be cost-effective as health interventions. Future research should focus on analysing tariffs for more finely grained processed food groups, including a particular focus on high-content sugar products and related obesity and overweight effects, which are well-known risk factors for CVD clinical outcomes (beyond compositional fatty acid intake effects).

Interestingly, we find evidence that Thailand is characterized by a second-best environment where efficiency gains can be reaped from increasing import tariffs on the “palm cooking oil” and “other edible oils” sectors. Whether or not this result extends to other major edible oil consuming and producing countries is an empirical question. In any case, our results suggest that, in the current context, edible oil fiscal food policies are either poor public health instruments due to a lack of potency driven by small trade shares (palm cooking oil) or not warranted due to adverse health impacts (other edible oils). We also note that, despite the strong recent fiscal food policy focus on limiting SSB consumption, beverages import tariffs turn out to be particularly cost-ineffective. For the same reason and due to small import shares, tariffs on SSBs are unlikely to be a potent public health instrument.

Despite limited cost-effectiveness, fiscal food policy tariffs generally tend to improve both nutritional, clinical health, demographic, and environmental indicators. Policy makers from Thailand and abroad, including WHO, would therefore do well to consider food sector tariffs, which could also serve other domestic policy purposes, as a potential intervention to maintain combined health and environmental sustainability of food consumption and production systems. Importantly, our results indicate that diet-related health improvements can go hand-in-hand with increased SFA intakes. The reason is two-fold: the literature suggests that SFA energy intake shares have moderate impacts on cholesterol build-up (Mensink et al., 2003) and intake shares of other unsaturated fatty acids may be affected more by tariff interventions. This is consistent with the evidence, found here, for Thailand, a middle-income country in nutritional and economic transition. Hence, while Thai policy makers have committed to implement WHO dietary guidelines, they (and other similar middle-income countries) would probably do well in considering the most appropriate approach to setting SFA targets to address unhealthy diets. In general, our fatty acid composition results suggest that WHO guidelines and policy indicators to address unhealthy eating (WHO, 2017b) could require some nuance, since it is not necessarily the reduction in SFA energy intake shares but the composition of fatty acid intakes which matters for reducing cholesterol-related CVD clinical outcomes. Furthermore, the WHO would do well in considering fiscal food policy interventions, generally, and import tariffs, more specifically, when drawing up lists of recommended interventions to reduce modifiable risk factors for NCDs.

## EA statement

This is to confirm that no ethics approval is required for our study.

## Footnotes

1 The six primary food crops include ‘Cereal grains’, ‘Oil palm, food’, ‘Coconut’, ‘Vegetables, fruit, nuts’, ‘Sugar cane’ and ‘Other crops’. The five processed food and beverage sectors include ‘Coconut oil’, ‘Palm cooking oil’, ‘Other refined vegetable and animal oils’, ‘Sugary foods’, ‘Other processed food products’, and ‘Beverages’.

2 Caregiver time losses for MI were considered small due to short illness duration, and therefore not included in the study. E.g. the 2010 GBD study only attributes MI disease burden to the first 28 days of illness (WHO, 2013).

3 The six primary food crops are: ‘Cereal grains’, ‘Oil palm for food’, ‘Coconut’, ‘Vegetables, fruit, nuts’, ‘Sugar cane’, and ‘Other crops’. The primary non-food crop is: ‘Oil palm for methanol’.
